# Bias in AI in Epic Electronic Medical Records and Its Impact on Executive Communication: Conceptual Analysis

**DOI:** 10.2196/85584

**Published:** 2026-07-31

**Authors:** Naomi Pemberton

**Affiliations:** 1 School of Health Sciences, Herbert H. and Grace A. Dow College of Health Professions, Central Michigan University, 1200 S Franklin St, Mount Pleasant, MI, United States, 1 917-701-4006

**Keywords:** AI, health care, algorithmic bias, AI in health care, Epic electronic medical records, executive communication

## Abstract

**Background:**

AI has become an essential component of modern health care delivery in Epic (Epic Systems Corporation) electronic medical record (EMR) systems, supporting predictive analytics, diagnostic decision-making, and population health management. Despite these advancements, evidence reveals that AI algorithms can perpetuate or even amplify existing health inequities through biased training data and flawed model design. Such algorithmic bias poses ethical challenges for health care leadership, regulatory compliance, and executive communication, especially in ensuring patient equity, transparency, and public accountability.

**Objective:**

This conceptual paper examines how algorithmic bias in Epic’s AI modules influences executive decision-making, organizational communication, and trust within health care systems. It integrates organizational communication theory and public health informatics research to propose a framework for ethical, transparent, and equitable communication in AI-integrated health care settings.

**Methods:**

Drawing upon the ethical communication and algorithmic trust framework (ECATF), this paper synthesizes interdisciplinary literature on AI bias, data governance, and leadership communication. The framework explains how transparent executive communication creates stakeholder trust in the context of bias identification and regulatory oversight.

**Results (Conceptual Findings):**

This conceptual analysis suggests that algorithmic bias influences leadership communication, equity framing, and governance strategies in AI-integrated health care systems. Incorporating AI bias auditing alongside regulatory monitoring and public education initiatives may support fairness, accountability, and health literacy across communities.

**Conclusions:**

As AI continues to shape health care leadership and policy, ongoing evaluation, ethical foresight, and regulatory vigilance are essential. Transparency, collaborative governance, and adaptive education will be necessary to ensure that AI supports equitable innovation rather than reinforcing unintended harm.

## Introduction

AI technologies are redefining health care operations, driving data-informed decision-making, and transforming how patient care is delivered. In Epic (Epic Systems Corporation) electronic medical record (EMR) systems, AI models are widely used for tasks such as predicting readmission risks, identifying care gaps, and optimizing clinical workflows. Examples include Epic’s predictive analytics and risk stratification tools, which are used to identify high-risk patients, monitor sepsis indicators, and support clinical decision-making. Although these tools promise efficiency, reduced administrative burden, and better patient outcomes, their use in health care systems has also raised concerns regarding bias, fairness, and ethical governance [1,2].

Algorithmic bias stemming from incomplete, unrepresentative, or historically biased data usually leads to unequal treatment recommendations, inaccurate risk stratifications, and disadvantages for marginalized populations. Such biases disproportionately affect racial and socioeconomic minorities, amplifying existing health disparities [3,4]. These concerns extend beyond technical accuracy because they influence how executives interpret AI-driven data, communicate organizational strategies, and shape institutional trust [5].

Health care leaders increasingly depend on AI-generated insights for strategic planning, performance evaluation, and policy formulation [6]. Yet, without a contextual understanding of data provenance and algorithmic limitations, executive communication may unintentionally reinforce inequities [7]. As AI systems become more embedded in executive decision-making, concerns regarding transparency, accountability, and trust become more urgent.

This paper examines the ways AI bias in Epic’s EMR systems influences executive communication and ethical decision-making practices. By integrating organizational communication theory, it explores how leadership framing, transparency, and relational trust can mitigate bias-related harm in health care organizations. Ultimately, this conceptual analysis underscores the need for equitable AI governance and communicative integrity in data-driven health care leadership.

## Methods

This conceptual analysis used a narrative literature synthesis to examine the relationship between algorithmic bias, executive communication, and ethical governance in AI-integrated health care systems. Literature published between 2018 and 2025 was identified through PubMed, Scopus, Google Scholar, and IEEE Xplore using combinations of keywords including “AI bias,” “Epic EMR,” “algorithmic trust,” “executive communication,” “healthcare AI ethics,” and “algorithmic transparency.”

Articles were included if they addressed health care AI, algorithmic fairness, organizational communication, trust in AI systems, or ethical governance in clinical or administrative health care settings. Priority was given to peer-reviewed journal articles and interdisciplinary scholarship addressing health care leadership and AI governance.

The selected literature was synthesized conceptually to identify recurring themes related to algorithmic bias, executive communication, transparency, and stakeholder trust. These themes informed the development and application of the ethical communication and algorithmic trust framework (ECATF).

## Results (Conceptual Findings)

The ECATF serves as the theoretical foundation for this paper because it links organizational communication theory with contemporary health informatics. Rooted in the principles of message framing, relational trust, and transparency, the ECATF explains how health care executives interpret, communicate, and act upon AI-generated insights in complex institutional ecosystems. The framework also highlights how communication mediates the relationship between algorithmic bias and stakeholder trust while emphasizing leadership’s responsibility to promote transparency and accountability in data-driven decision-making.

The framework consists of 4 interconnected components. In the ECATF, algorithmic bias may be operationalized through measures such as demographic disparities in prediction accuracy, false-positive or false-negative rates across patient populations, and documented bias audit outcomes. Executive communication transparency may be assessed through disclosure frequency, message clarity, stakeholder engagement, leadership briefings, and public reporting practices directed toward clinicians, patients, regulators, and community stakeholders. Trust may be evaluated through employee confidence surveys, patient trust perception measures, and stakeholder feedback regarding institutional transparency. These dimensions help explain how the ECATF functions across organizational communication and AI governance contexts [7].

The first stage is algorithmic bias identification. This stage involves recognizing and evaluating inequities embedded in Epic’s algorithms. It requires an understanding of how data inputs, training sets, and model parameters may inadvertently disadvantage certain populations. Effective bias identification requires collaboration among clinicians, data scientists, and communication professionals to address the technical and social dimensions of bias. The second stage is the executive communication response. Once bias is identified, leadership must determine how to frame and communicate these findings. Communication framing theory suggests that the way leaders communicate bias-related findings can either increase skepticism or strengthen stakeholder trust. When executives communicate openly about algorithmic limitations, acknowledge uncertainty, and explain corrective actions clearly, they are more likely to maintain credibility and preserve organizational trust. The third stage involves trust mediation. Trust operates as the central component of the ECATF. Communication practices shape both internal trust (among clinicians and staff) and external trust (among patients and the broader community). When leaders engage in open dialogue about algorithmic performance, limitations, and safeguards, they foster psychological safety and strengthen the institution’s integrity. Conversely, opacity or defensive messaging can amplify skepticism and weaken stakeholder relationships. The fourth stage is the equity feedback loop. This final component establishes ongoing mechanisms for transparency, bias correction, and stakeholder input. This feedback loop ensures that communication is not merely reactive but iterative, integrating lessons from audits, community feedback, and performance evaluations. The loop encourages continuous ethical reflection and adjustment of AI governance practices.

In this model, executive communication functions as a moderator and intervention point between AI bias and public perception. The ECATF thus argues that ethical communication is central to responsible AI governance. By institutionalizing transparent dialogue, consistent messaging, and equitable engagement, leaders can use communication to strengthen organizational trust and support equitable governance.

## Discussion

### Overview

This conceptual analysis highlights how algorithmic bias shapes leadership communication, policy responses, and equity-related decision-making. Bias in AI systems manifests through structural inequities, incomplete datasets, and technical limitations in algorithmic design. In Epic’s EMR platform, predictive models used for patient risk scoring, readmission forecasting, and clinical prioritization may yield skewed or inaccurate outputs across racial, gender, or socioeconomic groups [3,4]. These discrepancies reflect inequities embedded in the data infrastructure itself [3,7]. When biased AI outputs inform administrative decisions in areas such as funding allocation, staffing models, or quality improvement metrics, they can inadvertently perpetuate disparities in care delivery.

Health care executives who rely on AI-generated analytics to inform strategic and operational decisions often face the challenge of interpreting complex datasets without specialized expertise in machine learning or data ethics. Such a situation causes vulnerability, as algorithmic bias may go unrecognized or be misinterpreted. Consequently, effective executive communication is essential to bridge the divide between technical accuracy, ethical responsibility, and stakeholder understanding.

Recent scholarship, including the work of Lacmanovic and Skare [8], emphasizes the importance of systematic AI bias–auditing frameworks to promote fairness, transparency, and accountability in algorithmic systems. Their research highlights continuous model evaluation, stakeholder collaboration, and transparent reporting to support the development of equitable AI systems over time. Embedding such auditing mechanisms in Epic’s predictive models can enhance not only algorithmic reliability but also the clarity and credibility of leadership communication.

From a communication theory perspective, frameworks such as framing theory and relational trust theory help explain how health care leaders communicate uncertainty, limitations, and the steps they are taking to correct bias. Relational trust theory, meanwhile, suggests that open and empathic communication builds psychological safety in organizations and fosters credibility among external stakeholders [9]. These communication approaches enable executives to position AI as a tool that supports equitable and transparent health care practices.

Equally important is community education and engagement. Communication should extend beyond the institutional hierarchy to reach the patients and families who are most affected by AI-driven health care decisions. Community forums, digital literacy campaigns, and participatory governance models can improve public understanding of how AI-integrated health systems can enhance accountability and reinforce equity-centered leadership.

### Expanding the Conceptual Framework Beyond Epic

Although Epic is among the most widely used EMR systems globally, algorithmic bias is not unique to its platform. Similar ethical and communicative challenges exist across other digital health systems, including Oracle Health (formerly Cerner; Oracle Corporation) and Google Health AI (Google LLC). These systems rely on predictive modeling and large-scale data analytics to optimize care coordination, yet each faces comparable issues of transparency, model generalizability, and stakeholder trust.

The ECATF provides a framework for evaluating these challenges across diverse systems. For instance, Oracle Health’s clinical decision-support algorithms, which are used to flag deteriorating patients, have exhibited performance variation across demographic subgroups, prompting concerns about algorithmic equity. Likewise, Google Health AI’s diagnostic imaging models, despite demonstrating high technical accuracy, have faced scrutiny regarding data diversity and model explanation.

Applying the ECATF across these platforms allows leaders to adopt consistent communication protocols: explicitly identifying algorithmic limitations, explaining model updates, and inviting public and professional feedback. In doing so, organizations move beyond platform-specific problem-solving toward a unified model of ethical AI governance. The framework thus serves as a blueprint for strengthening transparency and trust across the broader digital health ecosystem, ensuring that technology development and leadership communication evolve in tandem toward shared equity goals.

### Practical Implications

This conceptual analysis highlights that bias auditing and ethical communication should function together in health care leadership. Executives must not only understand the technical foundations of AI but also develop competencies in transparent, audience-sensitive communication. Implementing periodic algorithmic audits, as recommended by Lacmanovic and Skare [8], ensures that model performance is continually assessed for fairness and accuracy. These audits should be accompanied by communication strategies that disclose audit findings and describe institutional responses.

From an operational standpoint, transparent reporting builds institutional credibility, fosters regulatory compliance, and strengthens interdepartmental collaboration [10]. Clear communication also plays a preventive role by reducing misinformation and improving alignment around accountability and inclusion.

At the community level, inclusive communication practices are equally vital. Public education campaigns that explain how algorithms influence care decisions and how biases are identified and corrected promote health literacy and trust [9,10]. Engaging clinicians, patient advocacy groups, and local communities in discussions about bias auditing democratizes and reinforces public engagement in AI governance. Participatory dialogue can help organizations maintain ethical oversight and human-centered AI governance.

### Limitations and Future Directions

As a conceptual paper, this study does not include empirical data or hypothesis testing. Instead, it establishes a theoretical foundation linking organizational communication with health informatics to address AI bias. Future research should adopt mixed methods designs, combining quantitative measures of trust and equity perception with qualitative interviews exploring executive communication strategies. Experimental and simulation studies could further evaluate how leadership framing influences clinician confidence and patient trust in AI-assisted care.

Longitudinal research can help assess how bias-auditing programs influence organizational transparency, equity outcomes, and public perception. Moreover, developing standardized metrics for communication effectiveness, such as message clarity, stakeholder engagement, and perceived fairness, would help operationalize the ECATF model across diverse health care contexts. Such empirical validation will enable health systems to translate ethical principles into more consistent communication and governance practices.

### Conclusions

AI has significant potential to improve health care efficiency, precision, and patient outcomes, yet it simultaneously presents complex ethical and communicative challenges. Algorithmic bias, if left unaddressed, can weaken trust and perpetuate inequity. The ECATF framework underscores transparent communication as the mechanism through which organizations sustain credibility and moral legitimacy.

To achieve ethical AI adoption, health care leaders must integrate bias auditing, data literacy training, and inclusive community engagement. These actions promote equity, public trust, and transparent governance in data-driven health systems. However, the long-term effects of algorithmic bias in health care are uncertain. As AI systems continue to evolve and influence clinical decision-making, the full scope of their impact, whether beneficial or detrimental, has yet to be evaluated.

While algorithmic tools can potentially enhance diagnostic accuracy, streamline operations, and personalize care, unchecked biases may also reinforce existing disparities or introduce new forms of inequity. Their effects can manifest over time, shaping care delivery patterns, patient outcomes, and institutional trust in ways that are difficult to predict or reverse.

Therefore, ongoing evaluation, ethical foresight, and regulatory review are necessary to understand the relationship between AI systems and human judgment. Future research should prioritize longitudinal assessments of AI bias and its influence on patient outcomes, clinical decision-making, and leadership communication. Transparency, collaborative governance, and adaptive education ensure that AI remains a force for positive transformation rather than a mechanism of inadvertent harm in health care.

Through sustained transparency, collaborative learning, and community partnership, health care organizations can ensure that AI remains an instrument of equity and ethical progress in an increasingly data-driven world.

## Abbreviations

ECATF: ethical communication and algorithmic trust framework
EMR: electronic medical record
